# Development and Molecular Investigation into the Effects of Carbamazepine Exposure in the Zebrafish (*Danio rerio*)

**DOI:** 10.3390/ijerph17238882

**Published:** 2020-11-29

**Authors:** Huihui Chen, Huiting Yang, Yanyan Zhao, Xiaohong Gu, Christopher J. Martyniuk

**Affiliations:** 1 State Key Laboratory of Lake Science and Environment, Nanjing Institute of Geography and Limnology, Chinese Academy of Sciences, Nanjing 210008, China; hhchen@niglas.ac.cn (H.C.); htyang305@gmail.com (H.Y.); yyzhao@niglas.ac.cn (Y.Z.); 2 State Key Laboratory of Developmental Biology of Freshwater Fish, College of Life Sciences, Hunan Normal University, Changsha 410081, China; 3 Inner Mongolia Key Laboratory of Environmental Pollution Control & Waste Resource Reuse, School of Ecology and Environment, Inner Mongolia University, Hohhot 010021, China; 4 Jiangsu Collaborative Innovation Center of Regional Modern Agriculture & Environmental Protection, Huaiyin Normal University, Huaian 223300, China; 5 Department of Physiological Sciences and Center for Environmental and Human Toxicology, University of Florida Genetics Institute, Interdisciplinary Program in Biomedical Sciences Neuroscience, College of Veterinary Medicine, University of Florida, Gainesville, FL 32611, USA; cmartyn@ufl.edu

**Keywords:** pharmaceuticals, carbamazepine, *Danio rerio*, gamma-aminobutyric acid, glutamate

## Abstract

Concerns regarding environmental exposures and the impacts of pharmaceuticals on non-target aquatic organisms continue to increase. The antiepileptic drug carbamazepine (CBZ) is often detected as an aquatic contaminant and can disrupt various behaviors of fishes. However, there are few reports which investigate the mechanism of CBZ action in fish. The aim of the current study was to evaluate the effects of CBZ on embryonic development (i.e., hatching rate, heart rate, and body length) and early spontaneous movement. Moreover, we sought to investigate potential mechanisms by focusing on the gamma-aminobutyric acid (GABA) neurotransmitter system in zebrafish 6 days after of exposure. The results show that CBZ exposure did not cause significant effects on embryo development (hatching rate, heart rate, nor body length) at the test concentrations. However, the early spontaneous movement of embryos was inhibited following 10 μg/L CBZ exposure at 28–29 h post-fertilization (hpf). In addition, acetylcholinesterase (AChE) activity and GABA concentrations were increased with exposure, whereas glutamate (Glu) concentrations were decreased in larval zebrafish. Gene expression analysis revealed that GABA and glutamate metabolic pathways in zebrafish larvae were altered following exposure to CBZ. GABA transaminase (*abat*) and glutamic acid decarboxylase (*gad1b*) decreased to 100 µg/L, and glutamate receptor, ionotropic, N-methyl D-aspartate 1b (*grin1b*) as well as the glutamate receptor, ionotropic, α-amino-3hydroxy-5methylisoxazole-4propionic 2b (*gria2b*) were down-regulated with exposure to 1 µg/L CBZ. Our study suggests that CBZ, which can act as an agonist of the GABA_A_ receptor in humans, can also induce alterations in the GABAergic system in fish. Overall, this study improves understanding of the neurotoxicity and behavioral toxicity of zebrafish exposed to CBZ and generates data to be used to understand mechanisms of action that may underlie antiepileptic drug exposures.

## 1. Introduction

Pharmaceuticals are bioactive chemicals used by humans for personal health and agricultural enterprises to improve animal growth [1,2]. Due to their widespread use, a significant number of pharmaceuticals have been discharged into aquatic environments [3,4,5]. Carbamazepine (CBZ) is one of the most frequently used antiepileptic drugs worldwide [6,7]. Due to its prevalence in water bodies and its resistance to removal during the sewage treatment process, CBZ is also one of the most frequently detected pharmaceuticals in aquatic environments [8]. CBZ has been detected in the range of 0.03 to 11.6 μg/L in various water bodies all over the world [9,10,11,12]. For example, CBZ was detected in 95% of the water samples collected from different locations around the United States of America (U.S.A.), and the mean concentration of CBZ in samples was ~350 ng/L [10]. Moreover, CBZ was detected and posed ecological risk at 20 different sites from the Baiyangding Lake and Taihu Lake areas in China, and the authors emphasized that more attention should be given to CBZ as an environmental contaminant [5]. Based on these and other studies, concerns regarding the impacts of CBZ on non-target aquatic organisms remain and warrant additional investigation.

Studies have demonstrated that CBZ can adversely affect non-target species in aquatic environments [6,7,13,14]. As vertebrates, fish are perhaps more susceptible to pharmaceutical exposure than invertebrates because pharmaceuticals are designed for human (vertebrate) use. Data on the toxic effects of CBZ on fish have been reported for different species, such as the zebrafish (*Danio rerio*), Chinese rare minnows (*Gobiocypris rarus*), and Japanese medaka (*Oryzia latipeus*) [15,16,17,18,19,20]. For example, 0.5 μg/L CBZ exposure increased embryo mortality, lowered plasma steroid hormone levels, and decreased egg production in zebrafish [21]. Moreover, 10 μg/L CBZ exposure can affect feeding behavior and can interfere with neurotransmission and the antioxidant system in zebrafish [16]. In addition, Yan et al. (2019) showed that CBZ may act as an endocrine disruptor in rare minnows [20]. Based on these studies, exposure to CBZ can adversely impact development, behavior and endocrine processes in fish. However, the underlying mechanisms of CBZ toxicity in fish are not well characterized.

Pharmaceuticals developed for human use often have well-established mechanisms of action (MoA), and as such, assessment of the effects of pharmaceutical drugs in aquatic animals should be based on their MoAs [22]. One of the MoAs of the antiepileptic drug CBZ in humans is related to the gamma-aminobutyric acid (GABA) neurotransmitter system [23]. GABA is the most abundant inhibitory neurotransmitter in the nervous system [24,25,26]. The GABA neurotransmitter system consists of several GABA receptor subtypes, each comprised of different subunits, as well as enzymes that synthesize and metabolize GABA [27]. Among the GABA receptors, GABA_A_ receptor α1 (Gabra1) plays a pivotal role in mediating rapid inhibitory synaptic transmission in the central nervous system [28]. The glutamate receptors, such as the “glutamate receptor, ionotropic, N-methyl D-aspartate 1b (Grin1b)” and the “glutamate receptor, ionotropic, α-amino-3hydroxy-5methylisoxazole-4propionic 2b (gria2b),” are also related to neurobehavior in fish and are associated with GABA signaling [29] as glutamate acts as the metabolic precursor for GABA synthesis. Glutamate decarboxylase (Gad1b) and GABA transaminase (ABAT) are the two main enzymes involved in regulating glutamate-GABA metabolic pathways [30]. Many antiepileptic drugs bind to GABA_A_ receptors to enhance GABAergic signaling to exert inhibitory actions in the brain [25]. CBZ is known to modulate the release, uptake, and receptor binding of neurotransmitters, and it acts as an agonist to the GABA_A_ receptor in humans [18]. However, most studies to date have not examined the MoA of CBZ in fish, and as a result, the potential ecological risks associated with CBZ may be underestimated.

As such, the effect of CBZ on the GABA neurotransmitter system in zebrafish was investigated in this study. Three nominal concentrations (1, 10, 100 μg/L) were used to study the toxicity of CBZ on zebrafish embryos. The lowest concentration targeted environmentally relevant levels of CBZ. The developmental toxicity of CBZ to zebrafish was first assessed, and several endpoints that included early spontaneous movement rate, hatching rate, heart rate, and body length were measured. Secondly, GABA and glutamate (Glu) concentrations, as well as transcript levels associated with GABA and glutamate receptor mRNA levels (*gabra1*, *grin1b,* and *gria2b*), and glutamate metabolic pathways (*gad1b* and *abat*) were determined in larval fish. Moreover, since acetylcholinesterase (AChE) is a widely used enzymatic biomarker of neurotoxicity and is often measured in studies to detect neurotoxic effects of various contaminants in the aquatic environment [31,32], AChE activity was also determined.

## 2. Materials and Methods

### 2.1. Fish and Culture Conditions

Adult zebrafish (wild-type AB strain) were cultured in a laboratory aquaculture system using aerated tap water. Fish were raised with a photoperiod of 14:10 h light: dark cycle and were fed with either live brine shrimp (*Artemia nauplii*) or a commercial fish diet on alternating days. For spawning, adult females and males (1:3) were placed into mating tanks. At 1–2 h post-fertilization (hpf), fertilized eggs from the same batch were examined by a stereomicroscope. The embryos that were developing normally were selected for subsequent experiments based upon staging outlined in Kimmel [33]. The fertilized eggs at shield stage (~6 hpf) were used for exposure experiments. All experimental procedures involving fish were approved by the Institutional Animal Care and Use Committee of the Nanjing Institute of Geography and Limnology, Chinese Academy of Sciences. At the end of the experiment, all zebrafish larvae were euthanized in liquid nitrogen, and those larvae that did not survive were collected and properly treated as hazardous wastes.

### 2.2. Exposure Proposal

CBZ (purity > 97%, J&K Chemical Ltd., Shanghai, China) was first prepared in amber vials as a stock solution (10 mg/mL) in dimethylsulfoxide (DMSO). The CBZ stock solution was prepared fresh each week. Selected embryos that were developing normally were exposed to CBZ. There were four treatments with the final concentrations of 0 (control, treated with 0.001% (*w*/*v*) DMSO), 1, 10 and 100 µg/L CBZ. The exposure solutions were prepared using the CBZ stock solution. The final DMSO concentration was 0.001% (*v*/*v*) in each group. There were two exposure experiments; one was conducted for development and gene expression, and a second exposure was conducted for enzyme-linked immunosorbent assay (ELISA) measurements. CBZ concentrations were quantified after the exposure solutions were dosed in the beaker or in the microplate each week according to our previously described method [6,14]. Briefly, for water samples, CBZ concentrations were quantified before and after water was refreshed. Water samples were filtered through 0.45 μm glass fiber filters and extracted using Oasis HLB cartridges (6 mL, 200 mg; Waters Corporation, Milford, MA, USA). CBZ was quantified using ultra-performance liquid chromatography tandem mass spectrometry X-TQD (UPLC–MS/MS; Waters Corporation, Milford, MA, USA).

#### 2.2.1. Exposure Experiment 1

Selected embryos from a single batch were randomly distributed among glass beakers (50 mL) containing 10 mL exposure solution, and there were 20 embryos in each beaker. There were five replicate beakers for each treatment. The final exposure solutions were refreshed daily (90% change) during the 6 d exposure experiment. During the exposure period, zebrafish embryos were placed in a constant temperature incubator, the temperature was maintained at 27 ± 1 °C, and the light dark cycle was 14 h:10 h (day:night). During the exposure period, 16 zebrafish embryos were randomly selected from each treatment group to measure their autonomous movement at 28–29 hpf. The hatching rate of all zebrafish embryos in each treatment group was counted at 48–62 hpf. The heart rate and body length of 12 and 10 zebrafish larvae in each treatment group were measured at 72 hpf and 96 hpf, respectively. After exposure, 5 larvae from each treatment group were collected and placed in a 1.5 mL centrifuge tube, frozen in liquid nitrogen, and stored at −80 °C for subsequent quantitative real-time PCR (RT-qPCR) analysis.

#### 2.2.2. Exposure Experiment 2

Exposure experiment 2 was carried out in a 12-well plate. Embryos (2400 embryos) were randomly transferred to 12-well Corning ultra-Low attachment microplates (Corning, NY, USA) (20 embryos per well) containing 4 mL of each exposure solution for 6 d, and 90% exposure solutions were renewed every 24 h. After 6 d exposure, 200 larvae in each treatment were collected and preserved as one biological replicate. Three biological replicates were constructed for each treatment (*n* = 3). After 6 d exposure, all the larvae were flash-frozen using liquid nitrogen and stored at −80 °C for GABA and Glu concentrations and AChE activity analysis.

### 2.3. Gene Expression Analysis

After 6 d exposure, the transcript levels of genes associated with the GABA neurotransmitter system and glutamate metabolic pathways, including *gabra1*, *grin1b*, *gria2b*, *abat,* and *gad1b,* were measured in the zebrafish larvae. RNA extraction, cDNA synthesis, and qPCR analysis were performed according to our previous methods [6]. Total RNA was isolated from larvae using TRIzol reagent (Invitrogen, Carlsbad, CA, USA). RNA quality and concentration were determined using BioTek Synergy HTX multi-function microplate (BioTek, VT, USA). RNA samples had a 260/280 nm absorption ratio ≥1.8 and these samples were deemed high quality for further analysis. cDNA was synthesized using the FastKing cDNA First Strand Synthesis Kit (Tiangen, Beijing, China) from 500 ng total RNA. Primers used in the study were obtained from previous literature [28,34]. The qPCR analysis was performed on the QuantStudio™ 3 Real-Time PCR System platform (Thermo Fisher Scientific, Carlsbad, CA, USA). The reaction system was as follows: a total volume of 20 μL, including cDNA samples, ROX Reference Dye (50X), TB Green^®^ Premix Ex Taq™ II (Takara, Japan) reagent, 200 nM upstream primer, and 200 nM downstream primer. The reaction conditions were as follows: after pre-denaturation, 40 cycles were performed at 95 °C × 5 s, 60 °C × 30 s. β-actin was used as the internal reference gene to normalize the expression of all target genes, and relative expression was calculated according to the delta-delta Ct method [35]. The primers and related information are shown in Table 1.

### 2.4. Measurement of AChE Activity, GABA, and Glu Concentrations

The AChE activity, GABA and Glu concentrations were measured using commercial ELISA kits following the manufacturer’s manual (No. MM-9110401, MM-9159901 and MM-9160601, respectively, Meimian, Jiangsu, China). Three biological replicates were conducted per treatment per assay.

### 2.5. Statistical Analysis

All data were analyzed using GraphPad Prism 6(GraphPad Software, San Diego, CA, USA). For developmental parameters and ELISA, a one-way analysis of variance (ANOVA) followed by Holm–Sidak’s multiple comparison test was employed to test for differences between mean values of treatments. One-way ANOVA followed by a Tukey’s multiple comparison test was used for gene expression data. All statistical data are expressed as mean ± standard error (standard error of mean, SEM), and *p* value < 0.05 was considered to indicate a significant difference among groups.

## 3. Results

### 3.1. Carbamazepine Quantification

CBZ concentrations in each treatment are shown in Table 2. No significant differences were observed between the nominal and tested values.

### 3.2. Early Spontaneous Movement and Development

**Figure 1:**
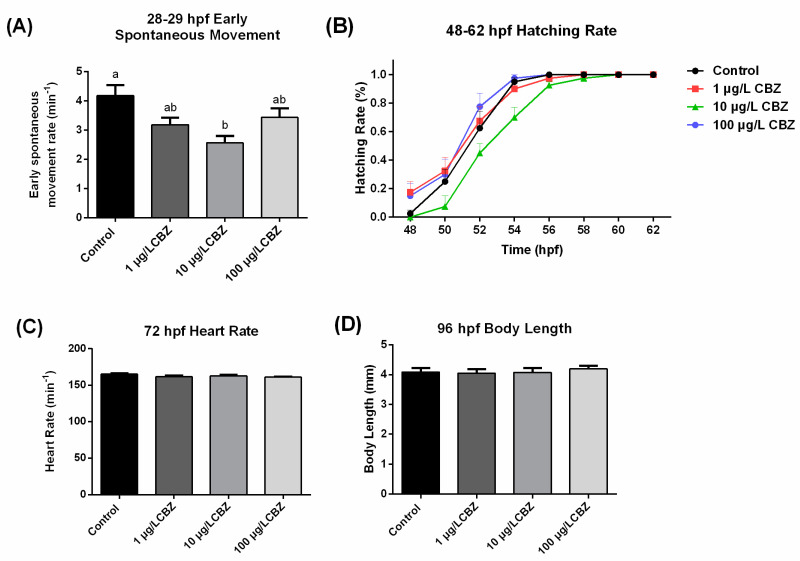
Carbamazepine (CBZ) effects on the development of zebrafish larvae after 6 d exposure (mean ± SEM). (**A**) Early spontaneous movement rate at 28–29 hpf (*n* = 16); (**B**) Hatching rate at 48–62 hpf (*n* = 50); (**C**) Heart rate at 72 hpf (*n* = 12); (**D**) Body length at 96 hpf (*n* = 10). Bars with different letters indicate significant differences between each other (*p* < 0.05).

No significant changes were observed in hatching rate, heart rate, or body length following exposure to CBZ (Figure 1B–D). However, the early spontaneous movement of embryos was significantly inhibited following exposure to 10 μg/L CBZ at 28–29 hpf (Figure 1A).

### 3.3. Gene Expression

**Figure 2:**
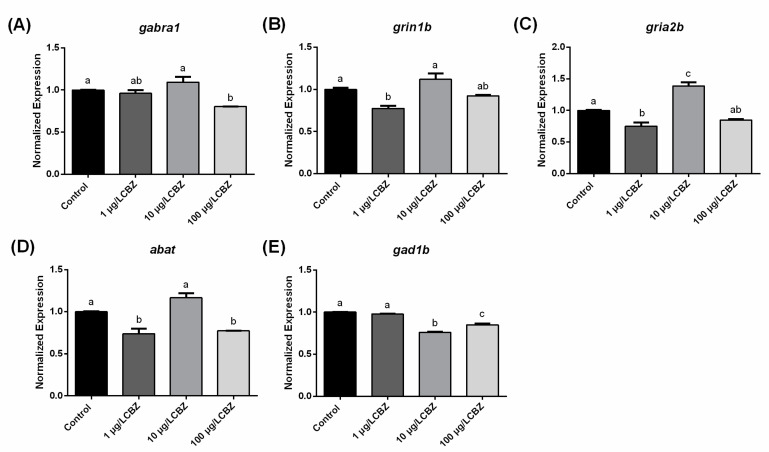
The steady state mRNA levels of (**A**) *gabra1*, (**B**) *grin1b*, (**C**) *gria2b*, (**D**) *abat*, (**E**) *gad1b* in zebrafish larvae following exposure to CBZ for 6 d (mean ± SEM). Bars with different letters indicate significant differences between each other (*n* = 5; *p* < 0.05).

The transcript level of *gabra1*, *grin1b*, *gria2b*, *gabra1*, *abat,* and *gad1b* were measured in zebrafish larvae following exposure to CBZ (1, 10, and 100 µg/L). Most of the genes were inhibited after CBZ exposure (Figure 2). The transcript level of *gad1b* was significantly downregulated in fish from both the 10 and 100 µg/L CBZ exposure groups; however, the *gabara1* transcript level was only reduced in fish from the 100 µg/L CBZ exposure group (Figure 2A,E). For *grin1b*, a significant reduction in transcript level was observed only in the 1 µg/L CBZ group (Figure 2B). The transcript level of *gria2b* was inhibited in fish from the 1 and 100 µg/L CBZ exposure groups, and a significant increase was observed in the 10 µg/L CBZ group (Figure 2C). The transcript level of *abat* was reduced in fish from the 1 and 100 µg/L CBZ exposure groups, and no significant differences were observed in fish from the 10 µg/L CBZ group (Figure 2D).

### 3.4. AChE Activity, GABA and Glu Concentrations Measurement

**Figure 3:**
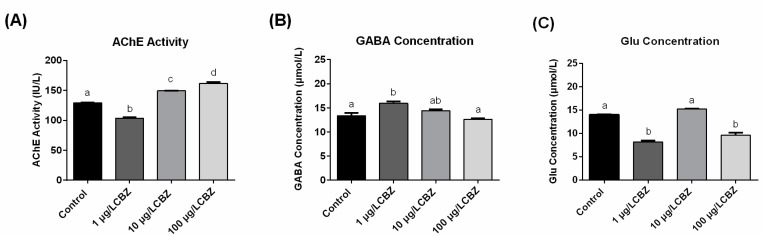
The acetylcholine (AchE) activity, gamma-aminobutyric acid (GABA) and glutamate (Glu) concentrations in the zebrafish larvae after 6 d of exposure to CBZ (mean ± SEM). (**A**) Acetylcholine activity; (**B**) Gamma-aminobutyric acid concentration; (**C**) Glutamate concentration. Bars with different letters indicate significant differences among groups (*n* = 3; *p* < 0.05).

After 6 d exposure, the AChE activity, as well as GABA and glutamate (Glu) concentrations in the zebrafish larval were measured (Figure 3). AChE was significantly increased in zebrafish larvae from both the 10 µg/L and 100 µg/L CBZ exposure groups (Figure 3A). Although no significant changes were observed with the highest concentration of CBZ, GABA concentration in the zebrafish larvae was significantly increased in larvae exposed to 1 µg/L CBZ exposure (Figure 3B). For Glu concentration, a significant decrease was observed in larvae from both the 1 and 100 µg/L CBZ treatment groups (Figure 3C).

## 4. Discussion

The current study aimed to investigate the effects of CBZ on the development, early spontaneous movement, and GABA neurotransmitter system in zebrafish larvae after 6 d exposure. Results showed that exposure to CBZ did not cause significant effects on the development of the zebrafish embryos at the concentrations tested. However, acute CBZ exposure induced alterations in AChE activity and the GABA neurotransmitter system in the zebrafish larvae, suggesting the potential for detrimental effects in the central nervous system during development.

Environmentally relevant concentrations of CBZ have been shown to affect fish more often during chronic exposures compared to acute exposures [14,36,37]. Acute toxicity tests have revealed that CBZ is unlikely to be lethal at environmental levels, with reported LC50 (median lethal concentration) and EC50 (median effective concentration) values approaching mg/L levels, and do not exert effects on major physiological processes, such as growth and activity [37]. For example, the 96 h LC50 values for CBZ in *O. latipes* is 35.4 mg/L [36], which is much higher than reported levels in the environment. The 72 h LC50 for CBZ in zebrafish embryo is ≥245 mg/L, and the NOEC (no observed effect concentration) for CBZ in zebrafish embryo on growth retardation has been reported to be above 30.6 mg/L [17]. In the current study, after a 6 d exposure to relatively low concentrations of CBZ (1, 10 and 100 µg/L), no significant changes in hatching rate, heart rate, or body length were observed. The present study agreed with previous studies, which determined that low exposure concentrations did not affect zebrafish growth [16]. However, the early spontaneous movements of zebrafish larvae were significantly inhibited following 10 µg/L CBZ exposure at 28–29 hpf. Our result agreed with a previous study [18] that CBZ can affect the early spontaneous movement of zebrafish. Moreover, similar to our study here, movement percentage and rest percentage of zebrafish larvae were significantly affected at lower levels of CBZ testing, while no effects were detected with the highest CBZ concentration [18]. Taken together, studies suggest that the effects of CBZ on the behavior of zebrafish larvae is non-linear nor dose-dependent. This may be related to a threshold between compensatory responses at lower levels of exposure and higher, potentially more toxic, concentrations. Nevertheless, studies should investigate further jobs and temporal specific responses to CBZ [38].

AChE hydrolyzes the neurotransmitter acetylcholine, acting to reduce synaptic transmission [39]. Unlike other neurotoxic compounds which can inhibit AChE activity, our study, in conjunction with previous investigations, demonstrates that CBZ can induce AChE activity in fish [16]. This discrepancy between CBZ and other neurotoxic agents may be related to specific mechanisms associated with CBZ. In response to an increase in AChE activity, a subsequent downregulation of acetylcholine receptors can occur [40]; this may explain, in part, the decrease of early spontaneous movement. It is worth noting that 10 µg/L CBZ had a significant impact on spontaneous movement and AChE activity, but no effect was observed at a higher dose (100 µg/L). The altered AChE activity in fish following exposure to pharmaceuticals may be related to the complex relationships between the cholinergic system and the exposure concentration [41]. For example, after exposure to another psychotropic drug, fluoxetine (50 and 200 µg/L), for 42 d, only one dose of those tested (50 µg/L) caused an increase of AChE activity following chronic exposure in *Pseudorasbora parva* [41]. Thus, the relationship between pharmaceutical exposure and AChE activity in larval fish is still unclear and may relate to the balance between therapeutic and toxic doses.

Excitatory (Glu) and inhibitory (GABA) neurotransmitters regulate activity and are involved in integrating signals from the periphery [28,42]. In the current study, the concentrations of GABA and Glu in zebrafish larvae were altered by CBZ exposure. One limitation of the current study is that it is not possible to isolate these effects specifically to the brain as whole larval fish were measured for neurotransmitter levels. Nevertheless, our data suggest that CBZ exposure may result in an imbalance between excitatory and inhibitory neurotransmission based upon altered receptor expression as well as biosynthetic enzymes. Taken together, changes in brain GABA are expected to contribute to the persistence of deficiencies in locomotor activity reported in studies using zebrafish [43,44,45].

As an agonist of the GABA receptor in humans [18], it is noteworthy to point out that CBZ significantly inhibited *gabra1* and *grin1b* transcript levels in the zebrafish larvae. This response may reflect alternative mechanisms of CBZ in fish compared to mammals, or it may be a compensatory response to over-activation of GABA_A_ receptors. GABA_A_ receptor activation is also involved in long-term potentiation and mediates connections that foster learning and memory [46]. The Gabra1 receptor subunit is localized at synapses in mature neurons and is structurally critical for the formation of ionic channels [47]. Recent studies have shown that GABA_A_ receptor expression is altered by environmental contaminants, such as phenazepam, which can also lead to abnormal regulation of feeding behavior [44]. Moreover, *abat* and *gad1b*, two enzymes involved in regulating glutamate-GABA metabolic pathways, were also inhibited by CBZ exposure. Glutamic acid decarboxylase 1 or Gad1b, is a major determinant of GABA levels. Gad1 is responsible for catalyzing the production of GABA from glutamate [48]. As reported, inhibition of Gad by (D,L)-allylglycine may lead to GABA depletion, seizures and neuronal damage in zebrafish [49]. As such, CBZ induced downregulation of GABA receptors and metabolic enzymes involved in the synthesis and degradation of GABA may lead to neurotoxicity and abnormal alteration in behavior in zebrafish.

To summarize, many endpoints measured here did not show a linear and dose-dependent effect. This may be due to the different pathways associated with pharmaceutical MoA or general toxicity of a contaminant. Pharmaceuticals have appropriate dosage and courses of treatment, and there is a balance between therapeutic effect and toxicity. It is reported that humans can respond to low doses of CBZ rapidly [50]. Here, we assessed toxicity of CBZ to zebrafish embryos/larvae, and a limitation is that a short exposure time was examined. Moreover, variability in biological response makes it challenging to link broad molecular, biochemical, and morphological endpoints together in a linear fashion. As such, the links that are made amongst endpoints should be interpreted with caution, and our goal here is to propose a working hypothesis for adverse responses in zebrafish to CBZ. However, each biological response may be independent from one another and may not involve interrelated mechanisms. Additional studies are needed to better define the relationship between GABA signaling and behaviors induced by pharmaceutical exposures.

## 5. Conclusions

In conclusion, our results suggest that exposure to low concentrations of CBZ can lead to decreased early spontaneous movement in zebrafish embryos. The increase in AChE activity is proposed to be related to these effects in zebrafish embryos but there are likely other mechanisms as well. Moreover, the modulation of the GABAergic neurotransmitter system at the transcript and metabolite level suggests that, as a human use antiepileptic drug that acts as an agonist of the GABA_A_ receptor in humans, CBZ can also induce alterations in the GABA neurotransmitter system in fish. Overall, this study improves understanding of the neurotoxicity and behavioral toxicity of fish exposed to CBZ, and generates data to be used to understand mechanisms of action that underlie antiepileptic drug exposures.

## Figures and Tables

**Table 1:** Primers used for gene expression analysis.

Symbol	Gene Name	Primer (5′-3′)	NCBI (National Center for Biotechnology Information) Accession Number
*β-actin*	Beta-actin	F: CGAGCAGGAGATGGGAACC R: CAACGGAAACGCTCATTGC	AF057040.1
*gabra1*	GABA_A_ receptor, α1	F: TCAGGCAGAGCTGGAAGGAT R: TGCCGTTGTGGAAGAACGT	NM_001077326
*grin1b*	Glutamate receptor, ionotropic, N-methyl D-aspartate 1b	F: CATGAGAACGGCTTCATGG R: GCCAGCTGCATTTGCTTCC	NM_001144131
*gria2b*	Glutamate receptor, ionotropic, AMPA 2b	F: ATGACAGTGACCGAGGAC R: CTTGAAAGAGTGAGCGATA	NM_131895
*abat*	GABA transaminase	F: GCGTTCAGGCAAAGCTCT R: GCAGGACGGAAACGGAT	NM_201498
*gad1b*	Glutamate decarboxylase 1b	F: AACTCAGGCGATTGTTGCAT R: TGAGGACATTTCCAGCCTTC	NM_194419

**Table 2:** Carbamazepine (CBZ) concentration (mean ± SD) measured in the exposure solutions.

Conditions	Control	1 μg/L	10 μg/L	100 μg/L
Nominal concentration (μg/L)	0	1.00	10.00	100.00
Measured concentration (μg/L)	No detected	0.90 ± 0.02	9.50 ± 0.22	92.80 ± 3.50
